# Competencies and training of radiographers and technologists for PET/MR imaging - a study from the UK MR-PET network

**DOI:** 10.1186/s41824-019-0070-6

**Published:** 2020-01-23

**Authors:** Marius Ovidiu Mada, Paula Hindmarch, James Stirling, James Davies, David Brian, Anna Barnes, Alexander Hammers, Nick Gulliver, Karl Herholz, John O’Brien, John-Paul Taylor

**Affiliations:** 1 University of Cambridge, Wolfson Brain Imaging Centre, Box 65, Adrian Way, Cambridge, CB20QQ UK; 2 Positron Emission Tomography Centre, Newcastle University, Building 15, Campus for Ageing and Vitality, Newcastle upon Tyne, NE4 6BE UK; 3 King’s College London, PET Centre, 1st Floor Lambeth Wing, St Thomas’ Hospital, London, SE1 7EH UK; 4 Invicro, Burlington Danes Building, Imperial College London, Hammersmith Hospital, Du Cane Road, London, W12 0NN UK; 5 University of Edinburgh, Edinburgh Imaging Facility QMRI, Queens Medical Research Institute, 47 Little France Crescent, Edinburgh, EH16 4TJ UK; 6 UCL Hospitals NHS Foundation Trust, 235 Euston Road, London, NW1 2BU UK; 7 Department of Nuclear Medicine & PET-CT, King’s College Hospital NHS Foundation Trust, Denmark Hill, London, SE5 9RS UK; 8 University of Manchester, Wolfson Molecular Imaging Centr, 27 Palantine Road, Manchester, M20 3LJ UK; 9 University Department of Psychiatry, University of Cambridge, Cambridge Biomedical Campus, Box 189, Adrian Way, Cambridge, CB20QQ UK; 10 Newcastle University, Institute of Neuroscience, Biomedical Research Building, Campus for Ageing and Vitality, Newcastle upon Tyne, NE4 5PL UK

**Keywords:** PET/MRI, Training, Competencies, Radiographers, Nuclear medicine technologists, Hybrid imaging practitioner

## Abstract

**Background:**

After the success of PET/CT as a clinical diagnostic tool, the introduction of PET/MRI is a natural development aimed at further improving combined diagnostic imaging and reduced ionising radiation dose for half-body imaging. As with PET and CT, the combination of PET and MRI presents a series of issues that need to be addressed regarding workforce training and education. At present, there is a lack of agreement over the competencies, training requirements and educational pathways needed for PET/MRI operation. In the UK, following the establishment of the MR-PET imaging network, a task force was created to investigate the status of the workforce training, identify gaps and make recommendations regarding staff training. To do this, we ran a national survey on the status of the workforce training and the local practices across the UK’s seven PET/MRI sites, reviewed the literature, and convened a panel of experts, to assess all the evidence and make recommendations regarding PET/MRI competencies and training of nuclear medicine technologists and radiographers.

**Results:**

There is limited literature available specifically on competencies and training for technologists and radiographers. The recommendations on the topic needed revisiting and adapting to the UK MR-PET network. The online survey confirmed the need for developing PET/MRI competencies and training pathways. Local organisational structures and practices were shared across the seven sites, based on models derived from experience outside the UK. The panel of experts agreed on the need for PET/MRI competencies and training strategies. Professional organisations started collaborative discussions with partners from both Nuclear Medicine and Radiography to set training priorities. Multidisciplinary collaboration and partnership were suggested as a key to a successful implementation of competencies and training.

**Conclusions:**

The report identified the need for establishing competencies for the PET/MRI workforce, particularly for technologists and radiographers. It also helped defining these competencies as well as identifying the demand for bespoke training and the development of local and national courses to be implemented to fulfil this new training need.

## Background

PET/MRI (Positron emission tomography/magnetic resonance imaging) is a relatively new hybrid imaging modality. Whilst primarily used in research, it is now transitioning towards becoming a clinical diagnostic tool. The first commercially available whole-body PET-MRI systems were installed in 2010 by Philips as two separated systems: a PET and MRI with the patient table moving between the two (Fendler et al., 2016). A year later, Siemens gained approval for the first simultaneous whole-body PET/MRI system (Beyer et al., 2018). By August 2017, there were about 130 systems installed worldwide including the newly Food and Drugs Authority (FDA)-approved general electric (GE) TOF (time of flight) PET/MRI (Beyer et al., 2018). According to the European Society for Hybrid, Molecular and Translational Imaging (ESHIMT)’s website, at the time of writing (September 2019), there are 62 systems installed in Europe alone (European Society for Hybrid Molecular and Translational Imaging, n.d.).

Although the clinical applications of PET/MRI are overlapping in many cases with the more mature PET/CT (positron emission tomography/computed tomography), there are recognised significant advantages of PET/MRI over PET/CT in Neurology, Cardiology, and Paediatric Oncology. The reduction in ionising radiation dose particularly for body imaging, superior parenchyma resolution, and additional functional and quantitative information are the main attraction to this modality (Nensa et al., 2014).

Of the neurological applications of PET/MRI, imaging diagnostics for dementia has become a major focus, and this reflects the UK Government commitment to fund dementia research (Department of Health, 2015). The Medical Research Council has funded five British universities to build PET/MRI facilities aimed at investigating dementias. These sites joined another two that operated PET/MRI services since 2012 and 2014, respectively, creating a network of seven PET/MRI sites (https://www.dpuk-mripet.info/member-sites). To help establish consolidated PET/MRI expertise in all the seven sites, funding was granted to a consortium of partners representing all institutions to address issues of communication, workforce training, performance harmonisation, and regulatory requirements for operation. Within this consortium, a training task force was established with the aim of reviewing current PET/MRI competencies, identifying any gaps, and making suggestions for competencies.

Establishing a harmonised framework for training to achieve agreed competencies for PET/MRI technologists and radiographers is crucial for securing a strong position for the network in the future, both in the UK and globally. This paper describes the work of this task force and presents a summary of its findings and recommendations regarding radiographers’ and nuclear medicine technologists’ competencies.

## Methods

We used four complementary methods to achieve our results. First, we undertook a review of the existing literature, including current published competencies and regulatory documentation. Secondly, we devised an online survey to scope and define training needs, which was then administered to members of the workforce in the seven imaging facilities. Thirdly, we contacted all seven PET/MRI centre leads to collate current training and competency requirements. Finally, we convened an interdisciplinary panel comprised of senior radiographers and nuclear medicine technologists (NMTs), educators, representatives of the professional societies, manufacturers, and PET/MRI clinical users and researchers. The panel was presented with all the collated evidence and then agreed on a number of statements pertaining to training requirements, needs, and gaps.

### Review of the literature

The search strategy for this paper is presented in Table 1.

**Table 1:** Search strategy used for the literature review

Database	Period	MeSH search	Results	Selected
PubMed	Jan 2012–May 2019	“PET/MR” [All Fields] AND (“education” [Subheading] OR “education” [All Fields] OR “training” [All Fields] OR “education” [MeSH Terms] OR “training” [All Fields])	21	3
		“hybrid imaging” [All Fields] AND (“education” [Subheading] OR “education” [All Fields] OR “training” [All Fields] OR “education” [MeSH Terms] OR “training” [All Fields])	22	5

Articles were selected as relevant based on their abstract and keywords.

In addition to the PubMed search, other resources for relevant publications were explored amongst the work of professional institutions active in the hybrid medical imaging community. These included Nuclear Medicine (British Nuclear Medicine Society) and Radiology (Royal College of Radiologists, Society and College of Radiographers) societies in the UK as well as equivalent European organisations (European Association of Nuclear Medicine and European Society for Hybrid, Molecular and Translational Imaging).

### The hybrid imaging training needs online survey

The hybrid imaging training needs online survey (HITNOS) was devised by the training task force of the Dementia Platform UK MR-PET Partnership to help assess the training status of staff working with PET/MR hybrid imaging, identify resources that may be suitable for all staff involved with PET/MR, identify gaps in training, highlight any barriers to training, and work with the network to address them. Ultimately, the survey was designed to facilitate the harmonisation of training in the PET/MRI facilities and improve awareness of, and access to, this training.

The survey was structured in eight sections: Introduction, Demographic information, Radiotracer capability, Past training, Other training relevant to PET/MRI, Barriers to training, Training needs, and Training delivery and schedule. The survey was distributed to the seven member sites of the DPUK Dementia Platform MR-PET Partnership between May and June 2017 and covered all categories of staff, all skills, and all levels of experience. The respondents were classified as (i) radiographers and nuclear medicine technologists, (ii) physicists, (iii) radiochemists, and (iv) non-clinical and (v) clinical researchers.

A complete description of the survey, including the results, is provided in Additional file 1.

### Local practices regarding training requirements and competencies for PET/MRI operation

Each site was invited to contribute to this exercise by providing their local practice documents, i.e. Standard Operating Procedures (SOPs) related to training, as well as a job description from their latest recruitment process for PET/MRI radiographers or technologists. The aim was to identify common practices regarding training requirements and competencies that could be used in drawing a consensus recommendation for the workforce training.

### The panel of experts

The main objective of this paper was to highlight the necessity of an agreement with regards to competencies, education, and training of the hybrid imaging workforce in general and the radiographers and NMTs in particular. Therefore, in constructing the panel of experts, the role of each group of professionals in the education and formation of the imaging practitioner was considered. The selection of the members was made so that it included radiographers and technologists active in the network, PET/MRI educational providers, professional organisations,, and the manufacturers. Additional file 2 presents the complete list of experts.

The panel of experts were provided with a report that assembled the findings from the literature review, the online survey, and the collection of local practices. Each then had time to review and make comments on the findings as well as suggestions for discussions. Items raised by each individual were collated, and a final version of the document, containing agreed conclusions from the panel, was agreed.

## Results

### Literature review

The subject of training and competencies for technologists and radiographers working in PET/MRI first appeared in published documents following the first installations of hybrid simultaneous scanners in 2012, initially in the USA (Gilmore et al., 2013). Both the nuclear medicine and radiology community identified the unique issues related to the operation of a PET/MRI scanner and put together a joint task force to analyse these issues and recommend a strategy for certification and educational requirements which was reported in 2013 by Gilmore et al. (2013). It was concluded that, at the undergraduate level, the curricula for both MRI and nuclear medicine technologists contain some information about the other modality but are already covering too many topics to allow more granularity. The solution to this problem would be a postgraduate course that would allow one imaging practitioner (technologist or radiographer) to operate the PET/MRI instead of the need for an MRI-qualified and a PET-qualified technologist, as is the situation in some sites.

Establishing the content of such a programme as well as the certification and the qualification of those supervising the training remains a difficult task. Nevertheless, there is consensus about the clinical competencies of the PET/MRI imaging practitioner. Gilmore et al. (2013) suggested the following:

- General patient care (to include MRI safety, radiation protection, and handling)
- Quality control of PET scanner and dispensing lab
- Patient preparation
- PET radiopharmaceuticals
- Basic physics and instrumentation of both MRI imaging and PET.

In the UK and parts of the rest of Europe, the approach to undergraduate training is slightly different, and the undergraduate radiography course contains some entry-level details about both PET and MRI, potentially making the pathway to a PET/MRI radiographer easier. There are nevertheless challenges in defining the competencies for PET/MRI radiographers.

The challenges of competencies and training in PET/MRI were also investigated amongst radiologists and nuclear medicine physicians. In a study from 2018, Beyer et al. (2018) analysed data from an international survey and found that the PET/MRI field was still developing and there were as yet no perfect combinations for training. The authors found that there was a drive for physicians to collaborate and learn from each other and maintain an up-to-date training record. Similar to findings from Gilmore et al. (2013), there was a trend towards a combined specialty and training curriculum.

The literature on other hybrid medical imaging modalities, like PET/CT, indicates the need for collaboration and cooperation between the nuclear medicine and radiology, both at national and international level. In the UK, in 2005, a consortium of radiologists, nuclear medicine physicians, radiographers, technologists, and clinical scientists published a document (Husband et al., 2005) that presented the strategy for development and integration of PET/CT. As well as presenting the requirements for radiographers’ and technologists’ workforce size, it also pointed out the need for training of nuclear medicine technologists in diagnostic CT and for training in radiation protection for radiographers. The authors acknowledged the good mix of skills brought in by the two professions and highlighted the need to further develop this in order to broaden the recruitment base. In addition, the document pointed out that appropriate training and accreditation must be provided to foster new opportunities for the radiographers and technologists, as PET/CT was rapidly developing as a clinical service.

In a document from 2006, the British Nuclear Medicine Society (BNMS) PET/CT Advisory Board (Marsden et al., 2006) listed the competencies for PET/CT radiographers or technologists. The experts formulated a list of underpinning knowledge that could either be incorporated in the undergraduate programme or a postgraduate course or be taught as a short intensive course lasting no more than one week. The practical experience could be achieved by a placement at an established site, under the supervision of a competent individual. They recommended that a minimum of 75 patients over the course of 4 months should be enough to demonstrate the clinical competencies.

The nuclear medicine technologists and radiographers play an important role in hybrid imaging, and this involvement can be challenging for their professional identity and training pathways. The case in the UK is particularly of interest as radiographers and technologists can be found working together in the same department, i.e. in a Nuclear Medicine Department, with a radiographer in a nuclear medicine technologist role. There is, however, a lag in the reciprocal recognition of nuclear medicine technologists as advanced practitioners, similar to radiographers. Gulliver and Hogg (2011) have expressed concerns that technologists might be left behind as the radiographer’s role expands further.

Griffiths (2015) tested the idea of “hybrid imaging workforce” as he identified, through interviews with experts in the nuclear medicine community, a series of challenges as well as opportunities. One of the challenges he identified is the risk of role erosion and automation that could lead to deskilling of the workforce and ultimately turning the profession away from the patient and towards the machine. One of the opportunities offered by hybrid imaging is the changes it makes to the workplace and patient workflow. In addition, over the course of a couple of decades, both technologists’ and radiographers’ roles developed, arguable at different rates. In the same paper, Griffiths (2015) propose the creation of a “hybrid imaging practitioner” who has a mix of traits derived from both modalities’ workforce and balances well the professional autonomy with the automated practice, delivering a patient-centric service. Only a clear career pathway for the technologists and radiographers working in hybrid imaging can support this ideal practitioner as well as an appropriate training and education framework. In addition to technical and clinical competencies, Griffiths’ work (Griffiths, 2015) suggests that humanistic competencies are necessary as well, including counselling and support for patients and psychological coping strategies for staff.

### Online survey results

One hundred six staff members from the seven UK PET/MRI sites were invited to participate in the survey. There are five main categories of staff that the survey was aimed at: (i) radiographers and technologists, (ii) physicists, (iii) radiochemists, and (iv) non-clinical and (v) clinical researchers. Sixty four individual responses were received equating to a response rate of 60%. In the case of the UK MR-PET network, five of the seven facilities were created mainly with a research focus.

**Fig. 1:**
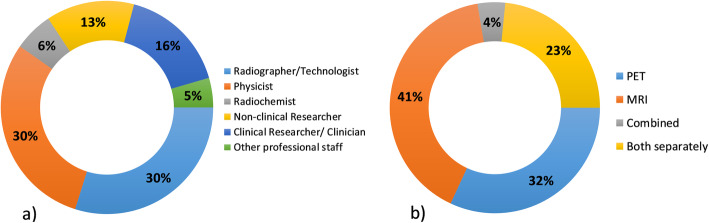
Distribution of respondents (**a**) according to their profession and (**b**) according to the experience with each modality

In terms of responses, there was an equal distribution of respondents between physicists, technologists/radiographers, and researchers. Similar to other facilities abroad (Beyer et al., 2018; Gilmore et al., 2013), there was a tendency for the staff working in PET/MRI to have an MRI background (42%), with slightly fewer staff members with PET experience (32%). Nearly a quarter of the respondents had experience in both modalities, but only 4% had prior experience in PET/MRI (Fig. 1).

**Fig. 2:**
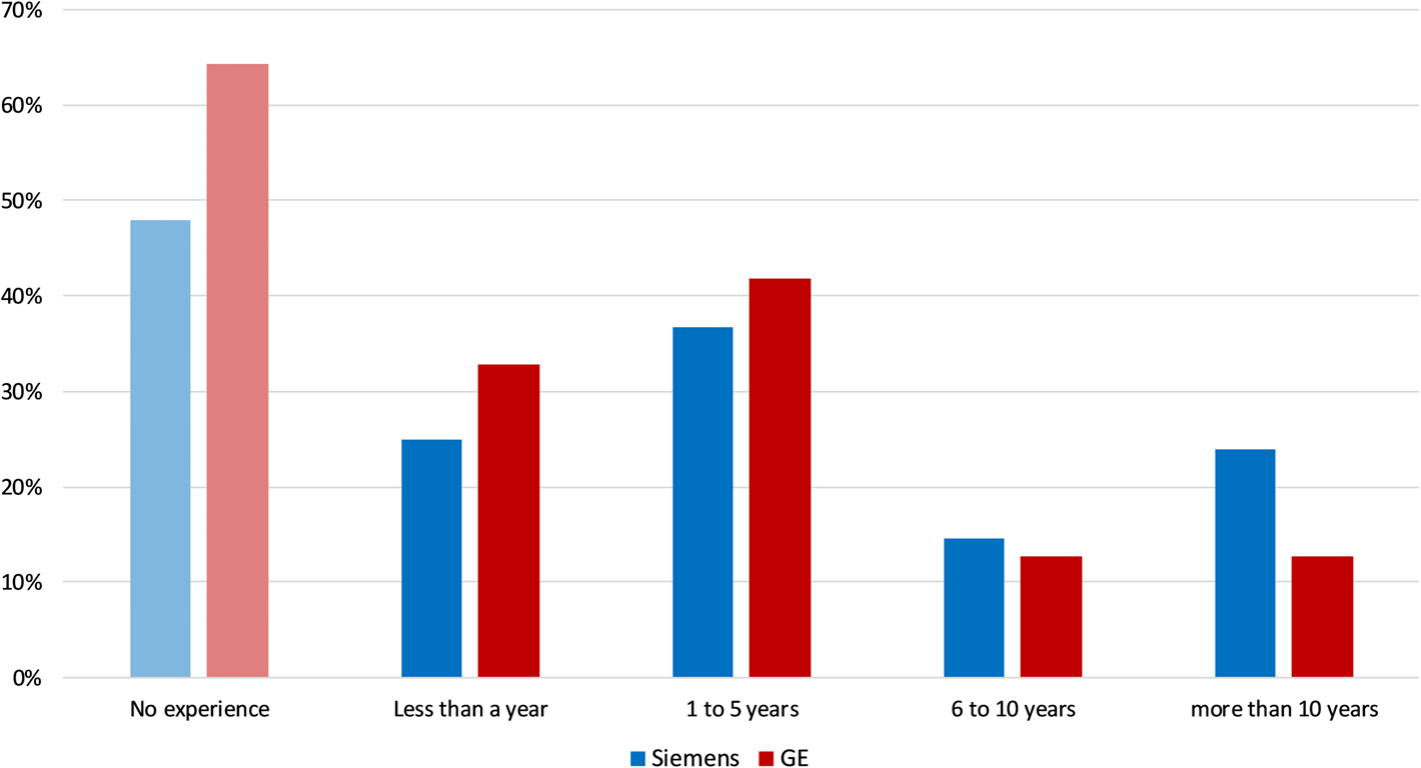
Distribution of respondents’ experience on operating machines by either manufacturer of the simultaneous PET/MR systems in the UK network

There are four GE SIGNA PET/MR systems and three Siemens mMR systems in the UK network. However, according to the survey, there was a tendency for the staff to be more Siemens trained; 48% had no experience with Siemens compared to 64% with no GE experience, and 38% of those who were familiar with Siemens had more than five years’ experience compared with 25% that had more than five years’ experience with GE systems (Fig. 2).

In the UK, radiographers must be registered with the Health and Care Professions Council (HCPC) to be allowed to practice. The Register of Clinical Technologists (RCT) sets the standards of the nuclear medicine technologist profession and records registered practitioners (The Register of Clinical Technologists (RCT), n.d.) but is not statutory, and registration of technologists is still voluntary.

Many staff members had had some sort of PET/MRI-related training in the three years before the survey, mostly arranged and delivered locally by senior colleagues or as part of the manufacturer’s scanner installation. All the current resources for training available for PET/MRI were highly rated. These resources are delivered as classroom lectures, hands-on sessions, or conferences in broadly equal measure.

**Fig. 3:**
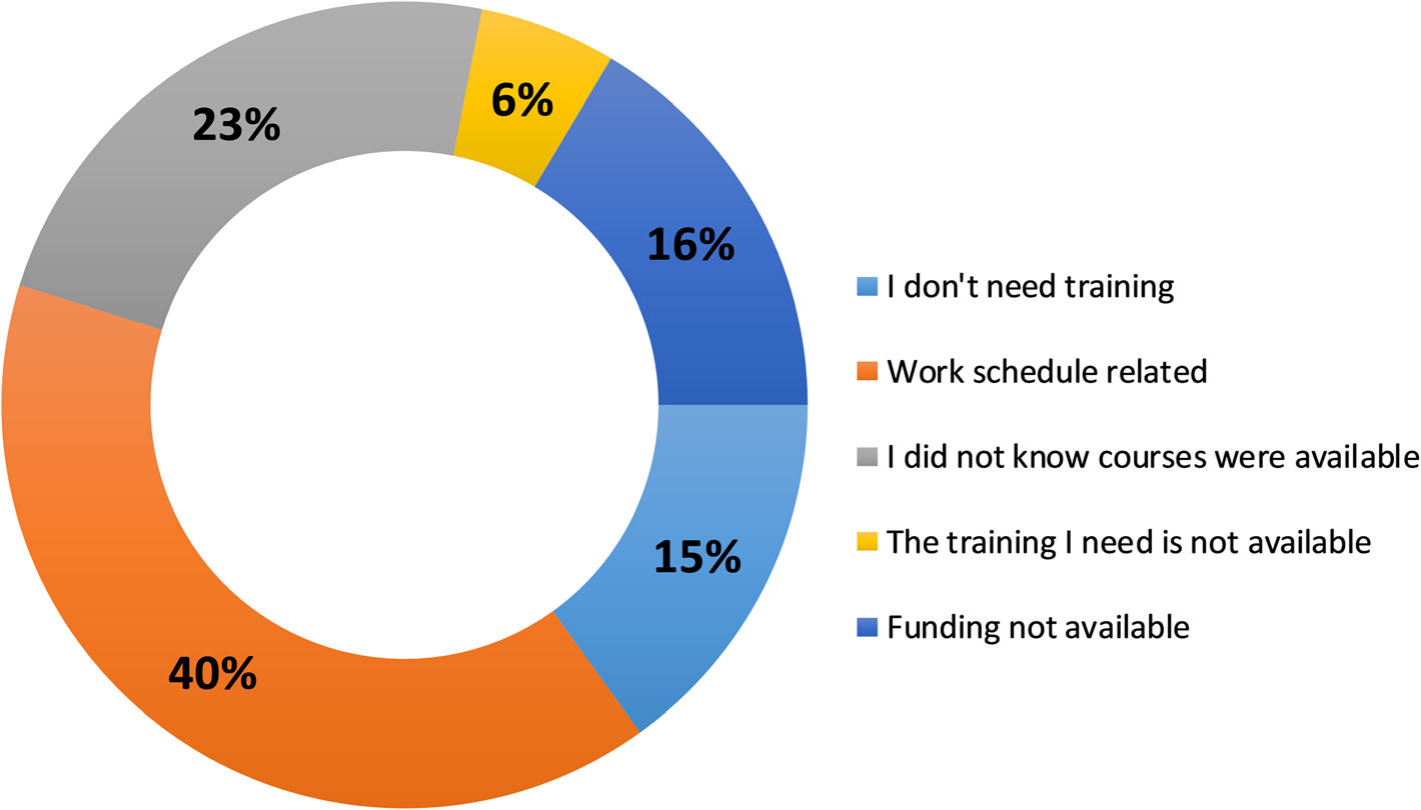
Barriers to training as identified from the online survey

The opinion of the respondents was that frequently, work schedule is preventing staff from attending training events. In addition, a quarter of the respondents were not aware of the training resources. Only 16% identified the access to funding as a barrier to training (Fig. 3).

### Local practices for PET/MRI competencies and training requirements

Two out of the seven members of the UK PET/MRI network had their instruments installed at least two years before the other scanners, putting them ahead in terms of establishing practices for competencies and training. At least four sites had employed radiographers or technologists as part of the PET/MRI installation and provided a copy of the job description for the advertised post. In addition, sites shared SOPs related to their training plan for radiographers and technologists.

Table 2 shows common competencies identified from the person specification from four of the seven sites at their last recruitment round for PET/MRI radiographer/technologist.

**Table 2:** Essential competencies listed in the Job Description of PET/MRI radiographers or technologists

Requirement	Essential
Qualifications & education	- BSc in Diagnostic Radiography or equivalent
	- Registration with HCPC or RCT
	- Postgraduate qualification in MRI or PET
Knowledge & experience	- Up-to-date Good Clinical Practice (GCP) training
	- Research-based practice
	- Experience in multidisciplinary teams
	- Knowledge and understanding of MRI safety
	- Knowledge and understanding of the Ionising Radiation (Medical Exposure) Regulations (IR (ME)R)
Skills & abilities	- IV cannulation
	- IT skills
	- Medical document writing skills
	- Interpersonal skills

Table 3 indicates the areas of competencies tested and the training required for operating the PET/MRI as per the local SOPs.

**Table 3:** List of competencies and training pathways

Competencies	Training
MRI safety	In-house MRI safety training
Radiation protection, e.g. use of, signage and monitoring, time/distance/shielding principles	Organised at departmental or institution/university level
Emergency procedures, e.g. quench, emergency stop	In-house MRI safety training
MRI coils	In-house MRI induction
Basic life support (BLS)	Organised at departmental or institution/university level
Manual handling/patient removal	Organised at departmental or institution/university level
Daily QC PET and MRI	In-house PET and MRI induction
Turning the scanner on and off	In-house PET and MRI induction
Basic operation of gantry and console	In-house PET and MRI induction
Patient table manual operation	In-house PET and MRI induction
Fire procedures	In-house Department induction
Equipment/plant room equipment	In-house Department induction
Patient monitoring	In-house PET and MRI induction
Contrast injector operation	In-house PET and MRI induction
Use of ancillary equipment	In-house PET and MRI induction

## Discussion and conclusions

PET/MRI continues to mature as a well-established multiparametric medical imaging modality. As more systems are installed across the globe there is mounting pressure on the workforce to adapt its practices to cope with the new challenges. By far, the most urgent issue when introducing a new imaging modality is adjusting the personnel training to meet the new requirements and preparing new graduates in line with these new competencies.

The introduction of PET/CT has had a dramatic effect on nuclear medicine practices, particularly on the role of radiographers and technologists, and led to new bespoke training courses being established (The Christie, n.d.). There has been considerable effort invested at national and international levels to cope with the fast integration of PET/CT in practice. In the UK, the PET/CT Advisory Board combined skills and knowledge from physicians, scientists, radiographers and technologists, and regulatory bodies to guide educators on how to train the workforce to enable this change as well as creating a profile of each role involved in the technique. Distance Assisted Training (DAT; Distance Assisted Training for Nuclear Medicine Professionals, n.d.) for Nuclear Medicine Professionals was a successful example of multinational and multidisciplinary collaboration for the benefit of the technologists. The online portal was later included in the International Atomic Energy Agency (IAEA) Human Health Campus (http://www-naweb.iaea.org/datol/).

The hybrid medical imaging family, of which PET/MRI is a member, has been of interest to the European healthcare community. Initiatives like the European Society for Hybrid, Molecular and Translational Imaging have created a forum to accelerate research in this field. However, there continues to be a lack of literature addressing the training and education of the PET/MRI workforce or documents to agree on necessary competencies. Nevertheless, since PET/MRI sits at the crossroad of multiple disciplines, there are research initiatives outside the PET/MRI mainstream that approach and discuss topics of interest for this issue. The case of the PET/MRI is unique but similarities with PET/CT exist and need investigating as some of the paradigms are shared with PET/MRI.

The UK initiatives from the early 2000s aimed at developing PET/CT services are a good example of multidisciplinary collaboration in hybrid medical imaging. In the case of PET/MRI, its clinical and commercial impact, as well as access to the technology remain relatively low compared to the quick adaptation of PET/CT, and initiatives like the PET/CT collaboration are less likely to be prioritised. However, the UK MR-PET network has already started to spark collaborations to establish training and accreditation of the PET/MRI workforce.

The PET/MR course from King’s College London and the postgraduate programme from the University of Edinburgh (Additional file 3) were developed to cover the theoretical aspects of PET/MRI without being primarily aimed at nuclear medicine technologists with no MRI experience or radiographers without nuclear medicine experience. The King’s course is offered as classroom lectures and the Edinburgh programmes are online. Funding to attend these courses is not as problematic as it was in the case of PET/CT as most of the seven PET/MRI sites are research facilities that have access to a broad range of funding sources, though this may be an issue for future purely clinical PET/MRI sites that might be established. These courses, like previous initiatives from the field of PET/CT (Distance Assisted Training for Nuclear Medicine Professionals, n.d.), lack accreditation by the professional regulatory bodies, i.e. HCPC or RCT. Higher education institutions (HEI) also struggle to finance postgraduate courses aimed at hybrid medical imaging modalities. This is an area that needs addressing and where the collaboration with the professional institutions will play a major part.

A similar approach to the one described for PET/CT by the BNMS PET/CT Advisory Board (Marsden et al., 2006) could be applied to PET/MRI. A panel of experts from the two communities would need to list the underpinning knowledge and the competencies specific to MRI and add them to the existing PET list. However, there are specific PET/MRI competencies, e.g. magnetic resonance-based attenuation correction (MRAC) quality assurance (QA) that need to be acknowledged and implemented using a different approach to the standard of combining individual MRI and PET competencies.

There are currently only seven PET/MRI facilities that operate in the UK and this could potentially limit the practical placements necessary to achieve the clinical competencies. There is however agreement, as indicated in the online survey, that shadowing of up to a week could be implemented easily and provide basic practical experience.

The situation in the UK is shared by the PET/MRI workforce internationally. In a series of workshops organised by the Tuebingen (Germany) PET/MR Group, lack of coordinated training appears repeatedly as a perceived barrier to PET/MRI progress (Bailey et al., 2018; Bailey et al., 2016). There is a clear need for expert input in the UK’s PET/MRI workforce development. Multidisciplinary teams and collaboration are essential to achieve the ideal workforce. There are good examples both from the USA (Gilmore et al., 2013; Delbeke et al., 2012) and from the experience of other UK hybrid imaging modalities. A conflict still exists over the “ownership” of the modality, but workforces stretching across the globe are working to minimise the negative effects of interdisciplinary conflict. In a paper from 2013, Beyer and Moser (2013) argue that combining two very complex physics in what we know as PET/MRI or MR/PET is a remarkable achievement and, besides the technical marvel, the combined clinical and research information is what should make this modality unique. They reiterate the need for collaboration and integrated professionals as well as combined training efforts. PET/MRI could provide more opportunities to close the gap between the extended role of the technologist and the radiographer in the context of hybrid imaging.

National PET/MRI networks like the one in the UK have the advantage of combining expertise from all members and the disciplines involved. Members have access to the latest research developments and have the potential to translate this into clinical practice at a faster pace. Radiographers and technologists working within the network have an important role in the dissemination of knowledge across the colleagues in the country and beyond.

The results of the survey are an important barometer of the UK PET/MRI community. They are in accordance with the results of Beyer et al. (2018) and open up the discussion about the competencies, particularly those for radiographers and technologists. The survey did not capture competencies directly, but it was important to clarify how the resources for training have developed in the absence of well-defined aptitudes for PET/MRI radiographers or technologists.

Establishing competencies for the radiographers and technologists working in PET/MRI and developing training and education resources to match these competencies must be driven by research and clinical demand. The imaging practitioners’ workforce has a pivotal role in defining the competencies for PET/MRI, and collaboration with all the stakeholders is crucial to the success of this venture. Educators and professional and regulatory institutions need to work together to devise courses that are accessible to technologists and are accredited. Lastly but equally important, the manufacturers need to engage with the PET/MRI community, including the radiographers and technologists, to refine the equipment in order to facilitate the translation of PET/MRI into clinical practice.

In conclusion, we have identified the need for defining competencies for acquiring simultaneous PET/MR images. In this report, we have outlined current practice in regard to the first seven PET/MRI sites in the UK, identified the common competencies required, and have outlined local and national courses which have developed to fill this need. We anticipate this report being of great value to new PET/MRI sites as they become established, whether primarily for research or clinical service use, and to PET/MRI networks in other countries as they develop training for this new hybrid imaging modality.

## Supplementary information

**Additional file 1.** The Hybrid Imaging Training Needs Online Survey – description and results

**Additional file 2.** Expert panel membership list

**Additional file 3.** PET/MR courses

## Data Availability

The datasets used and/or analysed during the current study are available from the corresponding author on reasonable request.

## Abbreviations

BNMS: British Nuclear Medicine Society
CT: Computed tomography
DAT: Distance assisted training
HCPC: Health and Care Professions Council
HEIs: Higher education institutions
HITNOS: Hybrid imaging training needs online survey
IAEA: International Atomic Energy Agency
MRAC: Magnetic resonance-based attenuation correction
MRI: Magnetic resonance imaging
PET: Positron emission tomography
QA: Quality assurance
RCT: Register of Clinical Technologists
SOP: Standard operating procedure
