# Supplementary material for: Competencies and training of radiographers and technologists for PET/MR imaging - a study from the UK MR-PET network
Source: Eur J Hybrid Imaging. 2020 Jan 23;4:1. doi: 10.1186/s41824-019-0070-6 (PMC6976550; doi:10.1186/s41824-019-0070-6)
Supplement: Supplementary file 1 — Additional file 1. The Hybrid Imaging Training Needs Online Survey – description and results [file 41824_2019_70_MOESM1_ESM.docx]

**Appendix 1. The Hybrid Imaging Training Needs Online Survey – description and results**

Introduction

The DPUK MR-PET Partnership Task Force 2: Training devised the Hybrid Imaging Training Needs Online Survey (HITNOS) to help assess the training status of staff working with MR-PET hybrid imaging, identify resources that may be suitable for all staff involved with MR-PET, identify gaps in training, highlight any barriers to training and work with the network to address them. Ultimately, the survey was designed to facilitate the harmonisation of training in the MR-PET facilities and improve awareness of, and access to, this training.

To achieve its objectives the survey aimed to sample all categories of staff, all skills and different levels of experience.

The HITNOS survey was designed using the Qualtrics online platform. This solution was chosen due to its capability to devise complicated survey structures and the tools for reporting and analysing the results.

The survey was distributed to the seven member sites of the DPUK Dementia Platform MR-PET Partnership between May and June 2017.

Survey design

The survey was structured in eight sections:

• Introduction

• Demographic Information

• Radiotracers

• Past Training

• Other training relevant to MR-PET

• Barriers to training

• Training needs

• Training delivery and schedule

Each section contained multiple choices questions with a section at the end to collect final remarks and comments.

There were general questions that applied to all respondents and specific questions aimed at particular categories of staff. To assure the integrity of data the respondent was required to answer each question displayed before moving further.

The survey was designed to be completed only once by each individual and could be stopped and resumed if necessary.

Distribution

An individual link to the survey was generated for each invited staff member. This had the advantage of allowing a better control of communication with responders and non-responders. Reminders were sent only to non-responders. The downside to this approach was that people could not share the link to facilitate the distribution of the survey to other colleagues not invited initially.

Sample size

It was not possible to equally distribute the number of invites to each site and category of staff due to lack of staff details at some sites (see Figure 1b). A total of 106 staff members from the seven member sites were invited to participate in the survey. They were identified either by referral by the contact person at each site and/or online research by the training team. Out of the 64 replies (60% response rate), three were incomplete and their data could not be used as their replies only included the first two questions. It was found that all three of these responders had used the same computer and the survey was not displayed properly in the web browser.

There are five main categories of staff that the survey was aimed at: i) radiographers and technologists, ii) physicists, iii) radiochemists, iv) non-clinical, and v) clinical researchers. Intrinsically, the proportion of researchers working in MR-PET facilities is higher than any other category of personnel and this was reflected in the distribution of invites (see Figure 1 a).

a)

b)

Figure 1. Distribution of invites a) according to their profession. RAD – radiographers and technologists, PHYS – physicists, RES – includes clinical and non-clinical, CHEM - radiochemists b) across the network and c) within each site. ED – Edinburgh, NCL -Newcastle, MAN – Manchester, CAM – Cambridge, UCLH – University College London Hospitals, KCL – King’s, IMP – Imperial.

c)

Results from completed questionnaires

Staff breakdown

a)

b)

Figure 2. Distribution of respondents a) according to their profession and b) according to the experience with each modality

Experience with the vendors’ platforms

Figure 3. Distribution of respondents’ experience on operating the two manufacturers’ platforms.

Professional registration of staff

Figure 4. Registration with regulatory bodies of MR-PET personnel. IPEM, IoP or other professional bodies are not regulatory so were excluded.

 Past training

Figure 5. Training attendance for the last three years across the staff groups.

Figure 6. Overview of main resources for training based on attendance. The dotted filled bars represent the percentage of respondents that attended the activity and the coloured bars shows the distribution of feedback on usefulness.

Other training relevant to MR-PET

There was a separate section in the survey to deal with resources that were not displayed in the list compiled by the authors of this survey. As with the compiled list, the aim is to capture how the training was delivered and how satisfied was the respondent with the learning outcomes.


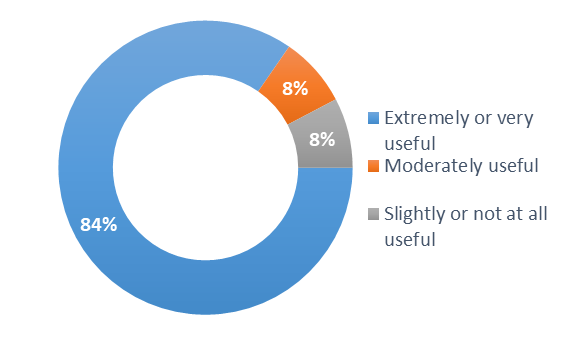

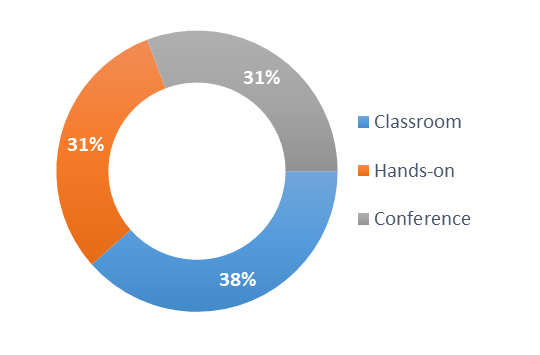


Figure 7. Other training relevant to MR-PET a) outcome satisfaction and b) delivery

Below there is a list of other training activities attended by the respondents:

• MRI in Dementia

• Work based learning

• Hands on local training

• GE PET/MR user meeting

• Hybrid Imaging course (Zurich)

• In house

• Pet reconstruction training

• IRMER

• SNMMI courses

• Cardiac MRI

• Several EU training seminars/workshops

• Explained basics on PET/MR methodology

• Courses in neuroanatomy

Barriers to training

Figure 8. Barriers to training of MR-PET personnel.

Budget for training

Figure 9. Distribution of training budge across sites, according to respondents.

he question of budget availability for training proved to be difficult to answer and this can also be explained by the variation in funding between the category of staff e.g. researchers tend to be funded by grants that will include funds for training as opposed to radiographers, for example, that most probably are not grant funded and hence rely on their department policy.

Training needs

Figure 10. Training needs as identified by the respondents.

Training delivery and schedule

Figure 11. Training delivery preferences.

There is a preference for face-to-face training activities i.e. Classroom, Hands-on and Conference, all three scoring more than 70% of the respondents (Figure 11). These findings are unexpected in the context of professional societies and organisations that are making efforts to facilitate access to online education and learning.


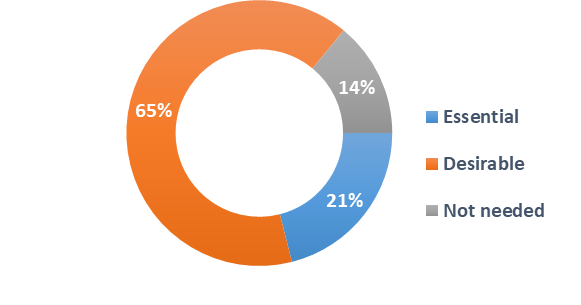

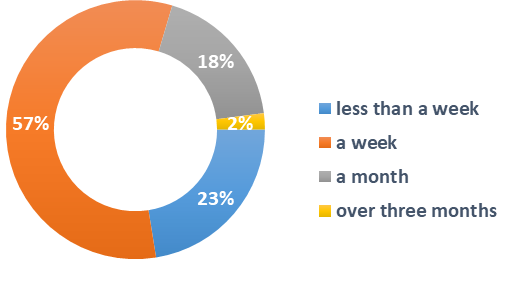


a) b)

Figure 12. a) Need for shadowing and for b) for how long.

Comments from respondents

The survey gave the opportunity for respondents to express their views on how the task force could collaborate better with the MR-PET community to achieve the set outcomes. Below is a summary of the comments left by the respondents.

The DPUK MR-PET Partnership Task Force 2: Training could help the MR-PET community by:

• Providing/maintaining and communicating a calendar for relevant courses, conferences and online training resources

• Supporting with funding and sign-posting individuals to funding resources

• Facilitating the sharing of experience/skills via online discussion board

• Organising local training

This section of the survey also collects general comments about the status of the community what other needs they might have.

Based on the replies received, the network needs:

• More practical knowledge

• To keep in touch with other radiographers to exchange ideas, experience

• To work on real projects in collaboration with experts

• To learn pulse programming

• Teaching to be organised locally

Conclusions

• The HITNOS survey was completed by 60% of respondents to whom it was sent (n=106). Importantly, there was representation from all the staff groups who we aimed to survey, and from all seven sites.

• Overall, the staff were an even mixture of at least MR and/or PET experience

• Majority of operational staff had training in the last three years

• Although overall there are more GE systems operating that Siemens, across the network there is more Siemens expertise. This situation is also reflected at individual site level where staff operating GE systems have just started using this platform with the arrival of the MR-PET

• Most of the staff have moderate experience (1-5 years of experience)

• Staff prefer local expertise and favour face-to-face learning, rather than on-line arrangements. Respondents were strongly in favour (86% essential or strongly desirable) of shadowing at other sites with an overwhelming preference (80%) for shadowing of a week or less.

• In general, staff have a busy schedule that prevent them from attending courses and funding is limited

• Existing courses are highly rated
