# Supplementary material for: Competencies and training of radiographers and technologists for PET/MR imaging - a study from the UK MR-PET network
Source: Eur J Hybrid Imaging. 2020 Jan 23;4:1. doi: 10.1186/s41824-019-0070-6 (PMC6976550; doi:10.1186/s41824-019-0070-6)
Supplement: Supplementary file 2 — Additional file 2. Expert panel membership list [file 41824_2019_70_MOESM2_ESM.docx]

**Appendix 2. Expert panel membership list**

**Radiographers**

James Stirling - PET/MRI Superintendent at King’s College London

James Davies - PET & MRI Research Technologist at Invicro London

Victoria Lupson - Superintendent Research Radiographer at University of Cambridge

Amy Watkins - Lead Research Radiographer (PET-MR) at University of Manchester

Paula Hindmarch - PET/MR Radiographer at University of Newcastle

David Brian - Superintendent Radiographer at University of Edinburgh

**Educational providers**

Prof Andrew Farrall - Honorary Professor of Neuroimaging & Education. Consultant Neuroradiologist. Director of the PET-MR principles & applications Cert, University of Edinburgh

Prof Alexander Hammers - Head of PET Imaging Centre and Professor of Imaging and Neuroscience, King’s College London & Guy’s St Thomas’ PET Centre

Dr Anna Barnes - Honorary Senior Lecturer at University College London and Clinical physicist at University College London Hospitals

**Professional organisations**

Nick Gulliver - Chair of the British Nuclear Medicine Society - Radiographers, Technologists and Nurses Group

Sue Johnson and Alexandra Lipton - Professional Officers at the Society and College of Radiographers

**Industry**

Dr Gaspar Delso - Senior scientist at GE Healthcare

Angela Meadows – Alliance Medical
