# Supplementary material for: Competencies and training of radiographers and technologists for PET/MR imaging - a study from the UK MR-PET network
Source: Eur J Hybrid Imaging. 2020 Jan 23;4:1. doi: 10.1186/s41824-019-0070-6 (PMC6976550; doi:10.1186/s41824-019-0070-6)
Supplement: Supplementary file 3 — Additional file 3. PET/MR courses [file 41824_2019_70_MOESM3_ESM.docx]

**Appendix 3. PET/MR courses**

**University of Edinburgh – PET/MR course syllabus**

1.0 Module: PET

1.1 Lecture: Principles of PET

1.1.1 Objective: Define PET imaging

1.1.2 Objective: Explain the radiotracer principle

1.1.3 Objective: Describe the physics & fundamental principles associated with PET imaging

1.2 Lecture: Acquisition & Reconstruction of PET

1.2.1 Objective: Identify and describe different acquisition protocols commonly used in clinical PET imaging

1.2.2 Objective: Explain basic principles of PET image reconstruction

1.3 Lecture: Principles of PET Quality Control

1.3.1 Objective: Explain the importance of quality control (QC) programmes in PET imaging.

1.3.2 Objective: Identify and describe routine QC procedures in PET imaging

2.0 Module: Radiopharmacy

2.1 Lecture: The Cyclotron

2.1.1 Objective: Describe the historical development of the cyclotron

2.1.2 Objective: Give an overview of the main cyclotron components & physical principles

2.1.3 Objective: List key safety considerations for the cyclotron

2.2 Lecture: Radiochemistry

2.2.1 Objective: Describe the principles of radiotracer creation

2.2.2 Objective: Describe the principles of radiotracer action

2.2.3 Objective: Discuss some common applications of radiotracers in PET scanning

2.2.4 Objective: List key safety considerations in the handling of radiotracers

3.0 Module: MR imaging techniques & physics

3.1 Lecture: MR Basics: basic principles behind MR

3.1.1 Objective: Describe "spin" and its relevance to Magnetic Resonance

3.1.2 Objective: Explain the relevance of protons in MR

3.1.3 Objective: Know the Larmor frequency equation

3.1.4 Objective: Describe “relaxation”

3.1.5 Objective: Define the “Free Induction Decay”

3.1.6 Objective: Distinguish between T1 & T2

3.2 Lecture: MR Basics: T1 & T2

3.2.1 Objective: Recognise different tissues have different T1 & T2 values

3.2.2 Objective: Understand how the differences are exploited to generate image contrast

3.3.3 Objective: Differentiate between T1 weighted & Proton Density weighted imaging

3.3.4 Objective: Understand what T1 imaging is useful for clinically

3.3.5 Objective: Understand T2 weighted imaging

3.3.6 Objective: Understand what T2 imaging is useful for clinically

3.3.7 Objective: Discuss how FLAIR & STIR imaging relate to each other

3.3.8 Objective: Know why FLAIR & STIR imaging are used

3.3 Lecture: MR Basics: Localisation

3.3.1 Objective: Explain MR slice selection

3.3.2 Objective: Describe how localization is performed in the MR image plane

3.3.3 Objective: Recognise the difference between frequency and phase encoding

3.3.4 Objective: State the difference between pixel and voxel

3.4 Lecture: MR Basics: k-Space

3.4.1 Objective: Explain what information lies in k-space

3.4.2 Objective: Describe how k-space relates to MR images

3.4.3 Objective: State the role of the Fourier Transform

3.4.4 Objective: List some common artefacts in MR images which result from errors and problems in k-space

3.5 Lecture: MR Health and Safety

3.5.1 Objective: Explain how to work in a high magnetic field safely

3.5.2 Objective: Describe differences in safety aspects of different types of MR scanner

3.5.3 Objective: List items which may cause hazard in a magnetic field

3.5.4 Objective: Discuss subject-specific factors that may affect safety

3.6 Lecture: Safe running of an MR unit

3.6.1 Objective: Outline the key factors involved in setting up and running an MR scanning facility for research in people

3.6.2 Objective: Describe how to ensure safety of staff and subjects or patients being scanned

3.6.3 Objective: Discuss current areas of debate concerning safety of magnetic fields and contrast agents

3.7 Lecture: Practical MR for Humans: screening for contraindications and safety

3.7.1 Objective: Describe the individual steps in preparing for an MR examination

3.7.2 Objective: Summarise the major contraindications to MR

3.7.3 Objective: Summarise the key things to watch out for to ensure safety

3.8 Lecture: Practical MR for Humans: having an MR scan

3.8.1 Objective: Explain what it is like to have an MR scan, from start to finish

4.0 PET-MR practicalities

4.1 Lecture: Introduction to PET-MR

4.1.1 Objective: Provide an overview of PET-MR applications

4.1.2 Objective: Discuss benefits & disadvantages of PET-MR compared to other imaging methods

4.1.3 Objective: Describe commercially available hybrid scanners & state the differences between them

4.2 Lecture: Attenuation correction of PET data using MR

4.2.1 Objective: Describe the basic physics principles of attenuation correction

4.2.2 Objective: List common problems & pitfalls of attenuation correction

4.2.3 Objective: Discuss applications of attenuation correction

4.3 Lecture: Patient handling & practicalities of PET scanning

4.3.1 Objective: Describe what constitutes appropriate facility set-up

4.3.2 Objective: Describe patient flow through a PET / PET-MR facility

4.3.3 Objective: Describe radiographer / technologist considerations in patient management

4.3.4 Objective: Describe scan / sequence set-up & execution

4.4 Lecture: Enrolment & consent of patients for PET-MR

4.4.1 Objective: Describe the core information required to be given to patients and volunteers

4.4.2 Objective: Discuss the key risks to be communicated to the subject (both MR and PET)

4.4.3 Objective: Describe the patient/volunteer preparation, with specific tracer requirements

4.4.4 Objective: Describe the consent process and how this relates to the specific examination

4.5 Lecture: Health & safety for PET-MR

4.5.1 Objective: Discuss the potential hazards of both PET & MR

4.5.2 Objective: Describe and put into practice the practical methods of minimizing hazards

4.5.3 Objective: Discuss the key legislation related to the modality and describe how these are put into practice

4.5.4 Objective: Describe the management of an adverse event which may occur

4.6 Lecture: PET-MR artefacts & pitfalls

4.6.1 Objective: Describe examples of differences in attenuation correction methods for PET-MR

4.6.2 Objective: Describe how coil attenuation may affect the image

4.6.3 Objective: Discuss common pitfalls in PET-MR imaging

4.7 Lecture: Case studies in PET-MR

4.7.1 Objective: Analyze & discuss situations which may arise in PET-MR scanning

4.7.2 Objective: Formulate solutions to situations which may arise based around case discussion

4.8 Lecture: Governance & standardisation in PET-MR

4.8.1 Objective: QA/QC

4.8.2 Objective: Patient communications

4.8.3 Objective: Radiation dose recording

4.8.4 Objective: Adverse event reporting

**King’s College London – Course on Simultaneous PET-MR: Science and Practice syllabus**

- MR basic physics, sequences, reconstruction, analysis; applications for brain, heart and cancer – **or:**
- PET basic chemistry, physics/ acquisition, reconstruction, analysis; applications for brain, heart and cancer
- Visits to either a PET or MR scanner, and then a PET-MR scanner
- History, current state-of-the-art instrumentation
- PET-MR specific MR sequences
- Practicalities
- Data correction (attenuation, motion)
- PET-MR specific image reconstruction and processing
- Reading of clinical scans with the experts
